# Familial Cancer and Prevention Molecular Epidemiology, A New Strategy Toward Cancer Control

**DOI:** 10.1054/bjoc.1999.0951

**Published:** 2000-01-18


*British Journal of Cancer* (2000) **82**(2), 499
© 2000 Cancer Research Campaign
Article no. bjoc.1999.0951

### Book Review

### Familial Cancer and Prevention Molecular Epidemiology, A New Strategy Toward Cancer Control

Edited by Utsonomiya, JJ, Mulvihill and W Weber
Publication details,

This book is a publication of the proceedings of the International Union Against Cancer (UICC) Symposium 'Familial Cancer and Prevention Molecular Epidemiology, A New Strategy Toward Cancer Control' held in Kobe, Japan, in 1997. Although the meeting was held 2 years ago, the book is extremely up-to-date and indeed contains much new information which is of great interest for the specialist in familial cancer. In the preface, the editors state that the UICC strategy of controlling cancer through genetics could be summarized in the term 'ASURA'. In Buddhism, 'ASURA' is a national treasury with three arms and three faces which symbolizes strong power against cancer, the ability to control through genetic testing and a faithful focus for ethical, legal, psychological and social issues. The editors have retermed ASURA as an *A*dvanced *S*ymposium for *U*niversal *R*engineering *A*gainst *C*ancer. This sums up the aim of this very interesting book. This is definitely a book for the specialist in the field and contains much new and interesting information for professionals already managing individuals with a family history of cancer. It is not a book for the beginner in this field. The book takes a very holistic approach to the management of familial cancer and includes papers from experts in basic laboratory science through to those involved in clinical management and also includes papers on ethics and psychosocial issues. These encompass papers from individuals in many countries to try to provide a world-wide perspective.

There is very good, comprehensive coverage of many of the current controversies and uncertainties in the field and the collection of papers from the symposium are collated into sections which are easy to follow.

An example of the diversity and holistic approach of inclusion of papers within this book is that, in the second section after the introduction, there are papers regarding logistics of setting up a family cancer clinic (although many of the papers are highly specialized), this is followed by counselling issues, including issues covering views from different countries as diverse as the USA and Japan, through to specialized clinical management issues in colorectal cancer, the controversial role of prophylactic colectomy in the Lynch syndrome and the scientific basis of genetic testing in this syndrome.

Some knowledge of cancer genetics is needed to obtain maximum benefit from this book. This is a very thought-provoking book and many of the papers are at the cutting edge of the current field. This text would be a very useful addition to any oncology or genetics library and is essential reading for the cancer genetic specialist.

*Rosaling Eeles*
*Institute of Cancer Research*
*Sutton*
*Surrey*
*SM2 5NG, UK*

